# Investigation of the Prevalence of High-Risk Human Papillomavirus, Human Herpesvirus-8, and Herpes Simplex Virus-2 in Cervical Biopsy Samples Using the Real-Time PCR Method

**DOI:** 10.3390/tropicalmed10070200

**Published:** 2025-07-17

**Authors:** Ayfer Bakır, Betül Yüzügüldü, Eylül Beren Tanık, Muhammed Furkan Kürkçü, Gizem Korkut, Firdevs Şahin Duran

**Affiliations:** 1 Department of Microbiology, Ankara Etlik City Hospital, University of Health Sciences, Ankara 06170, Türkiye; betulsomuncu09@gmail.com (B.Y.); eyllbrn92@gmail.com (E.B.T.); furkankurkcu@gmail.com (M.F.K.); gizemkosak@gmail.com (G.K.); 2 Department of Pathology, Ankara Etlik City Hospital, University of Health Sciences, Ankara 06170, Türkiye; firdevssahinduran@yahoo.com

**Keywords:** human papillomavirus infection, HHV-8, HSV-2, histopathology, real-time PCR

## Abstract

Persistent high-risk human papillomavirus (HR-HPV) infection is closely associated with the development of cervical intraepithelial neoplasia (CIN) and cervical cancer. In recent years, the potential impact of viral co-infections on this process has also been investigated. This study investigated the presence of HR-HPV, HSV-1/2, and HHV-8 DNA in formalin-fixed paraffin-embedded (FFPE) cervical biopsy samples, as well as their association with lesion severity. A total of 276 FFPE cervical tissue samples were evaluated. Viral DNA was detected by real-time PCR. The samples were histopathologically classified as normal/non-dysplastic, low-grade (LSIL), and high-grade (HSIL) lesions. HR-HPV DNA was detected in 112 samples (40.6%), with the highest prevalence observed in the 30–39 age group (51.2%). Among the HPV-positive cases, 46.5% (52/112) had single-type infections, 32.1% (36/112) had multiple-type infections, and 21.4% (24/112) were untypable. Together, these categories accounted for all HPV-positive samples. The most common genotype was HPV-16 (16.7%). HHV-8 and HSV-2 DNA were not detected. HSV-1 DNA was detected in only three non-dysplastic, HPV-negative cervical samples. In conclusion, HR-HPV DNA was detected in 40.6% of cervical biopsy samples and showed a significant association with increasing histological severity, highlighting its critical role in the progression of cervical lesions. Although the absence of HHV-8 and HSV-2 suggests a limited contribution of these viruses to cervical disease, the use of a single real-time PCR assay limits the ability to draw generalized conclusions regarding their clinical relevance. Further large-scale, multicenter studies employing both tissue-based and serological approaches are needed to validate these findings and to better understand the dynamics of viral co-infections in cervical disease.

## 1. Introduction

Cervical cancer is one of the most common gynecological malignancies and a leading cause of cancer-related deaths among women. High-risk human papillomavirus (HR-HPV) infection is the primary etiological factor in both cervical intraepithelial neoplasia (CIN) and cervical cancer, with persistent HR-HPV playing a critical role in the progression from CIN to invasive disease [1,2]. Approximately 40 HR-HPV types are known to affect the genital tract and account for more than 99% of all cervical cancer cases reported, most of which are from developing countries [3,4].

In recent years, viral co-infections such as human immunodeficiency virus (HIV), cytomegalovirus (CMV), herpes simplex virus type 1 and 2 (HSV-1/2), human herpesvirus 6 and 8 (HHV-6/8), and Epstein–Barr virus (EBV) have been suggested to amplify HPV’s oncogenic potential by impairing local immunity, inducing chronic inflammation, and promoting cellular transformation [5,6,7,8]. Furthermore, sexually transmitted infections like HIV, hepatitis B virus (HBV), and hepatitis C virus (HCV) may facilitate HPV persistence by compromising host defenses, increasing the risk of cervical cancer [9,10,11].

Recent epidemiological studies have highlighted the potential roles of HHV-8 and HSV-2 in cervical carcinogenesis, suggesting that these viruses may promote HPV persistence and disease progression through mechanisms such as chronic inflammation, mucosal barrier disruption, and local immunosuppression. In vitro evidence further indicates that HSV-2 can enhance HPV oncogene expression, while HHV-8 may support HPV-driven neoplastic changes by inducing cytokine production and vascular alterations [6,12,13,14,15,16].

HHV-8 (Kaposi sarcoma-associated herpesvirus, KSHV) is an established cause of Kaposi sarcoma, primary effusion lymphoma, and multicentric Castleman disease, but its interaction with HPV in cervical tissue remains unclear [12]. Reported HHV-8 DNA detection rates in cervical specimens vary considerably, from 0% to 22.9%, depending on the anatomical site and detection method [13]. Similarly, HSV-2, primarily known for causing genital ulcers, may facilitate HPV and other sexually transmitted infections by compromising mucosal integrity [13,14]. Although not directly oncogenic, HSV-2 has been associated with persistent HPV infection through chronic inflammation and immune modulation [15,16]. Its role in high-grade lesions or invasive cervical cancer remains controversial [16,17,18,19,20].

Reliable identification and typing of high-risk HPV are essential for guiding both clinical care and broader public health strategies [21]. Although formalin-fixed paraffin-embedded (FFPE) cervical tissue is commonly used in molecular studies, due to its long-term stability and availability [4,22], studies exploring viral co-infections in these samples remain scarce, and most rely on serological data, which may not accurately reflect viral DNA in cervical tissue [6,13]. In contrast, molecular techniques applied to FFPE specimens offer a more direct and reliable means of detection. However, the potential contribution of viral co-infections, such as HHV-8 and HSV-2, to the progression of cervical lesions remains unclear, and data from FFPE cervical tissues are particularly limited.

Therefore, this study aims to investigate the presence of HR-HPV, HHV-8, and HSV-2 DNA in FFPE cervical biopsy specimens of different histopathological grades and to assess the frequency of co-infection between HR-HPV and HHV-8 to better understand the potential role of these viruses in cervical neoplasia.

## 2. Materials and Methods

### 2.1. Study Design and Sample Collection

This study was designed as a retrospective, cross-sectional analysis. A total of 276 archived cervical biopsy specimens, preserved as FFPE tissue blocks, were retrieved from the Pathology Laboratory of Ankara Etlik City Hospital. These specimens had been submitted by the gynecology outpatient clinic between 1 November 2022 and 30 June 2023.

Inclusion criteria consisted of samples from women aged 18 years or older who had been histopathologically examined and reported, and for whom complete demographic and clinical data were available in the hospital information system. Samples were excluded if patients were younger than 18 years, if the tissue had not undergone histopathological reporting, if relevant clinical data were missing, or if the FFPE material was unsuitable for molecular analysis, due to inadequate preservation or degraded DNA quality.

To minimize selection bias, all eligible samples submitted consecutively within the specified time frame were included without randomization or selective filtering. This consecutive sampling strategy ensured comprehensive representation of cervical lesions encountered in routine clinical practice during the study period. Although no formal sample size calculation was performed beforehand, the study aimed to include the entire pool of qualified archival cases within the defined period to ensure representativeness. This appropriate approach aligns with the exploratory objectives of the study.

For each FFPE block, at least three serial tissue sections were prepared. If DNA yield or quality from the first section was insufficient for PCR amplification, DNA extraction and PCR were repeated using the second and, if necessary, the third section from the same block. Using this strategy, all included samples yielded adequate DNA for downstream molecular analyses. After applying these criteria, a total of 276 eligible cases with complete histopathological and molecular data were included in the final dataset. After reviewing the hematoxylin and eosin (H&E)-stained slides to confirm histopathological adequacy and diagnosis, a board-certified pathologist selected the corresponding FFPE tissue blocks for molecular investigation.

### 2.2. Ethical Consideration

Ethical approval for this study was granted by the Clinical Research Ethics Committee of Ankara Etlik City Hospital (Approval Date: 9 August 2023; Decision No: AEŞH-EK1-2023-390). The study adhered to the principles of the Declaration of Helsinki.

### 2.3. Histopathological Evaluation

**Figure 1:**
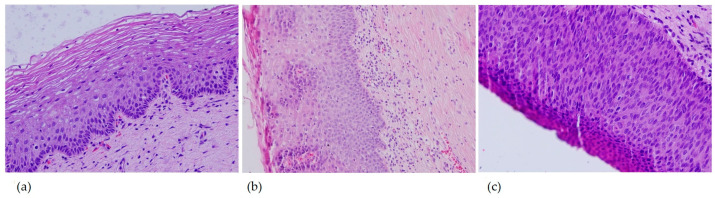
Representative histopathological images of cervical biopsy specimens (Hematoxylin & Eosin stain, original magnification ×20). (**a**) Normal/non-dysplastic cervical squamous epithelium (**b**) Low-grade squamous intraepithelial lesion (LSIL) (**c**) High-grade squamous intraepithelial lesion (HSIL).

All cervical biopsy specimens were reassessed independently by an experienced pathologist using light microscopy. Lesions were categorized into three groups according to standard diagnostic criteria: normal/non-dysplastic cervical epithelium, low-grade squamous intraepithelial lesion (LSIL), and high-grade squamous intraepithelial lesion (HSIL) (Figure 1). Classification followed the guidelines of the World Health Organization (WHO) and the 2014 Bethesda System.

Histological features associated with HPV-related changes, such as the extent of dysplasia, nuclear atypia, mitotic activity, and the presence of koilocytosis, were evaluated during the diagnostic process.

### 2.4. DNA Extraction and Sample Preparation

From each selected FFPE block, 5 µm-thick sections were cut using a sterile microtome and transferred into 1.5 mL screw-cap Eppendorf tubes using sterile toothpicks. Deparaffinization was performed with 1 mL of xylene (Merck, Darmstadt, Germany) at 45 °C for 15 min, followed by centrifugation at 14,000 rpm. The xylene wash was repeated once. The pellet was then washed twice with 98% ethanol.

After ethanol evaporation, DNA was extracted using the Zybio EXM3000 automated system (Zybio Inc., Chongqing, China) and the Biospeedy^®^ Rapid Nucleic Acid Extraction Kit (Bioeksen R&D Technologies, İstanbul, Türkiye) according to the manufacturers’ instructions. Extracted nucleic acids were stored at −20 °C until further analysis.

### 2.5. Detection of HR-HPV, HHV-8, and HSV-1/2 DNA

Detection of HR-HPV, HHV-8, and HSV-1/2 DNA was performed using commercially available real-time PCR kits (Bioeksen R&D Technologies, İstanbul, Türkiye): Human Papilloma Virus qPCR Test Kit (Cat. No: HRHPV-Screen-T-25), Human Herpes Virus 8 qPCR Test Kit (Cat. No: BS-HHV8-25), and Human Herpes Simplex Virus 1/2 qPCR Test Kit (Cat. No: BS-HSV-25). Amplification was conducted on the Magnetic Induction Cycler (Mic)-PCR system (Bio Molecular Systems, Upper Coomera, QLD, Australia) following the manufacturer’s instructions.

The HPV assay targeted the E1-E7 and L1 genomic regions, detecting 14 high-risk genotypes. Genotype-specific identification was provided only for HPV-16, -18, and -45, whereas the remaining high-risk genotypes—HPV-35/52, HPV-33/51/59, HPV-39/56/68, and HPV-31/58/66—were detected in pooled categories, without resolution to individual types. These untypable results, therefore, reflect high-risk genotypes detected within these pooled groups, which are beyond the assay’s genotype-specific identification capability, rather than falling below detection thresholds.

HHV-8 DNA detection targeted the ORF73 (LANA) gene region, whereas HSV-1 and HSV-2 were identified using primers targeting the US5′ (HSV-1) and US3′ (HSV-2) regions, respectively. Positive and negative controls were included in each PCR run. Amplification curves and cycle threshold (Ct) values were interpreted using the software provided with the system. 

The Ct cut-off value, limits of detection (LoD), sensitivity and specificity values, and internal amplification control information for each assay are summarized below:

HPV qPCR assay: Ct cut-off ≤ 26; LoD: 2000–4800 copies/mL; sensitivity: 99.5%; specificity: 98.48%. Internal amplification control targeting the RNase P gene was included.

HSV-1/2 qPCR assay: HSV-1: Ct cut-off ≤ 26; LoD: 96.4 copies/mL; sensitivity: 100%; specificity: 98.64%. HSV-2: Ct cut-off ≤ 26; LoD: 88.6 copies/mL; sensitivity: 99.68%; specificity: 97.89%. Internal amplification control targeting the RNase P gene was included.

HHV-8 qPCR assay: Ct cut-off ≤ 26; LoD: 188.85 copies/mL; sensitivity: 100%; specificity: 99.68%. Internal amplification control targeting the RNase P gene was included.

In all assays, the internal control was used to monitor for PCR inhibition and to ensure the validity of negative results.

### 2.6. Statistical Analysis

Statistical analysis was performed using IBM SPSS Statistics version 25.0 (IBM Corp., Armonk, NY, USA). Descriptive statistics were presented as frequencies, percentages, medians, and interquartile ranges (IQRs). The Pearson chi-square or Fisher’s exact test was used to compare categorical variables, as appropriate. For continuous variables, the Mann–Whitney U test was employed. The correlation between HPV positivity and increasing histopathological severity (for example, LSIL and HSIL) was assessed using Spearman’s rho test. To evaluate the independent associations between age, HR-HPV genotypes, and lesion severity (LSIL/HSIL), a multivariate binary logistic regression analysis was performed, and the results were expressed as odds ratios (ORs) with 95% confidence intervals (Cls). A *p*-value < 0.05 was considered statistically significant.

## 3. Results

### 3.1. Patient Demographics and HPV Prevalence

The median age of the 276 women included in the study was 44 years (IQR: 37–53), with an age range of 30 to 82 years. HPV DNA was detected in 112 of 276 cervical biopsy samples (40.6%, 95% CI: 35.0–46.5%). Neither HHV-8 nor HSV-2 DNA was detected in any of the samples. HSV-1 DNA was detected in only three cases (1.1%).

The median age of HPV-positive patients was 43 years (IQR: 36–49), significantly younger than the HPV-negative group, whose median age was 46 years (IQR: 39–57) (*p* = 0.002). HPV prevalence declined with increasing age: 51.2% in the 30–39 group, 44.0% in 40–49, 35.0% in 50–59, and 17.9% in those aged ≥60 years (*p* = 0.004). The age-specific distribution of HPV positivity is presented in Table 1.

### 3.2. HPV Genotype Distribution and Infection Patterns

Among the 276 patients, 52 (18.8%) had single-type HPV infections, 36 (13.0%) had multiple-type infections, and 24 (8.7%) carried untypable HPV types. When considering only the 112 HPV-positive samples, 52 (46.5%) had single-type infections, 36 (32.1%) had multiple-type infections, and 24 (21.4%) were untypable. These categories together accounted for all HPV-positive cases.

Age group differences in these infection patterns were statistically significant (*p* = 0.009). The median age of patients with single infections was 44 years (IQR: 37–52), while those with multiple infections had a median age of 37 years (IQR: 32–44) (*p* = 0.002).

HPV-16 (16.7%) and HPV-18 (2.2%) were the most commonly identified single genotypes. The most prevalent multiple-infection pattern involved co-infection with HPV-35/52 and HPV-33/51/59 (4.3%) (Table 2).

**Figure 2:**
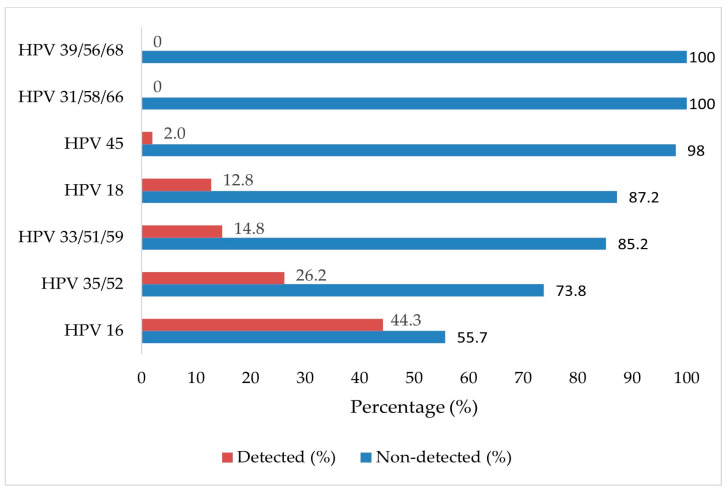
Distribution of high-risk human papillomavirus (HR-HPV) genotypes detected in cervical biopsy samples (n = 112).

A total of 149 HPV genotypes were identified in 112 HR-HPV-positive cases, owing to the presence of multiple infections. HPV-16 was the most frequently detected genotype, observed in 66 instances (44.3% of all genotypes identified). This frequency reflects the total number of genotype detections, not the number of individual patients. This was followed by HPV-35/52 (26.2%, n = 39), HPV-33/51/59 (14.8%, n = 22), HPV-18 (12.8%, n = 19), and HPV-45 (2.0%, n = 3). The distribution of HPV genotypes is presented in Figure 2.

### 3.3. Histopathological Correlations

Of the 276 cervical biopsy specimens, 99 (35.9%) showed premalignant lesions, classified as either LSIL or HSIL. LSIL was the most common lesion type (18.1%, n = 50), followed by HSIL (17.8%, n = 49). No case of cervical carcinoma was observed.

HPV DNA was significantly more prevalent in premalignant lesions (91.9%, 91/99) compared to normal or non-dysplastic histologies (11.9%, 21/177) (*p* < 0.001). Among HPV-positive cases, HSIL was the most frequently associated histological outcome (95.9%, 47/49), followed by LSIL (88.0%, 44/50). In the normal/non-dysplastic group, only 11.9% (21/177) were HPV-positive.

**Figure 3:**
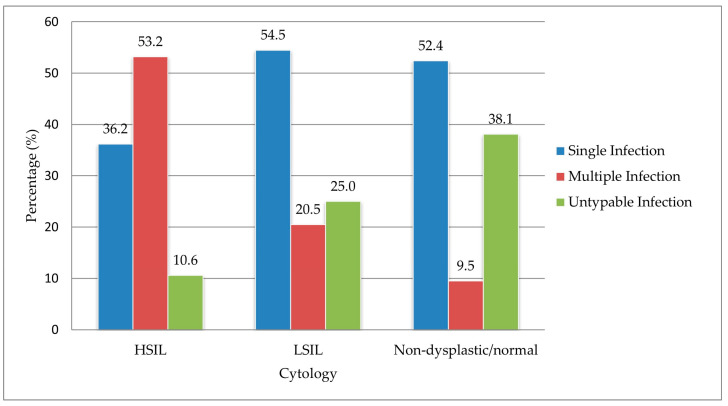
Patterns of single, multiple, and untypable high-risk human papillomavirus (HR-HPV) infections across histological diagnoses.

Of the 112 HPV-positive cervical biopsy samples, 16.8% (n = 21) exhibited normal/non-dysplastic histology, 39.3% (n = 44) were classified as LSIL, and 42.0% (n = 47) as HSIL. Single infections were most frequently detected in the LSIL group (54.5%, 24/44), followed by the normal/non-dysplastic group (52.4%, 11/21), and the HSIL group (36.2%, 17/47). Multiple infections were most commonly observed in HSIL samples (53.2%, 25/47), followed by LSIL (20.5%, 9/44), and normal/non-dysplastic tissues (9.5%, 2/21). Figure 3 illustrates the distribution patterns of single, multiple, and untypable HPV infections across the different histological classifications.

Single-type infections were significantly more frequent in premalignant lesions (41.4%, 41/99) compared to normal/non-dysplastic samples (6.2%, 11/177) (*p* < 0.001). HPV-16 was the predominant genotype detected in both LSIL (42%) and HSIL (32.7%) categories (Table 2).

### 3.4. Association Between HR-HPV Status, Age, Infection Pattern, and Cervical Lesion Severity

HR-HPV positivity across different age groups was not found to be a statistically significant risk factor for having LSIL or HSIL compared to normal/non-dysplastic histology. The OR for other age categories, when the 30–39 age group was used as the reference, are presented in Table 3.

The relative risk of having LSIL and HSIL histological diagnosis was found to be 84.5 times higher in HPV-positive women compared to HPV-negative women (OR = 84.5; 95% CI: 35.96–198.57, *p* < 0.001).

Among women with multiple HPV infections, the risk of LSIL and HSIL was 4.56 times higher compared to those with single HPV infections (OR = 4.56; 95% CI: 0.95–22.00, *p* = 0.059). In contrast, no significant association was observed between untypable HPV infections and cervical lesion severity (OR = 0.54; 95% CI: 0.18–1.58, *p* = 0.258) (Table 3).

The correlation between HPV positivity and increasing histopathological severity (for example, LSIL and HSIL) was assessed using Spearman’s rho test.

### 3.5. Herpesvirus Detection

No HHV-8 or HSV-2 DNA was detected in any of the cervical biopsy samples. HSV-1 DNA was identified in three cases (1.1%), all of which were from patients with normal/non-dysplastic histology. One patient was aged 40–49, and the remaining two were in the 50–59 age group. All three individuals were of Turkish origin.

No co-infection involving HR-HPV, HHV-8, or HSV-2 was observed.

## 4. Discussion

In this study, HR-HPV DNA was identified in 40.6% of FFPE cervical biopsy samples. HPV positivity was significantly more frequent in premalignant histopathologies (91.9%) compared to normal or non-dysplastic histologies (11.9%) (*p* < 0.001), which supports the well-established role of HR-HPV in the pathogenesis of cervical lesions [2,3,23]. Among the genotypes detected, HPV-16 was the most common in both LSIL and HSIL cases, consistent with its previously reported high oncogenic potential [24,25,26]. HPV detection was most frequent in women aged 30–39 and then declined gradually in older age groups, a trend also observed in earlier epidemiological studies [23,27].

Multiple HPV infections were notably more prevalent in younger patients and in high-grade lesions, which may be attributed to increased viral exposure or to age-related differences in immune response [24,28]. Co-infection with several HR-HPV genotypes may increase the likelihood of developing cervical intraepithelial neoplasia (CIN) and may hinder viral clearance [28].

Although many studies have reported that multiple HPV infections are a significant risk factor for high-grade cervical lesions and may exert a synergistic effect on the development of precancerous lesions such as LSIL and HSIL [29,30], there are also reports indicating that multiple HPV infections do not have a synergistic effect on high-grade cervical lesions [31]. In the present study, infections involving multiple HPV types were found to carry a higher relative risk for LSIL/HSIL lesions compared to single-type HPV infections.

Regarding the association between age and the risk of developing high-grade cervical lesions, previous studies have generally reported no significant relationship [29,31]. Similarly, in this study, increasing age among HPV-positive individuals was not found to pose a significant risk for high-grade lesions, which is consistent with the existing literature.

From a public health perspective, the frequent detection of HPV-16, HPV-18, and HPV-45 reinforces the utility of existing vaccines that target these high-risk genotypes. Notably, other high-risk genotypes, such as HPV-35/52 (26.2%) and HPV-33/51/59 (14.8%), were frequently identified after HPV-16. These findings may support the case for future vaccine formulations that provide broader genotype coverage [21,23,24].

Previous studies have proposed a potential role for herpesviruses, particularly HHV-8 and HSV-2, in cervical carcinogenesis, primarily through mechanisms such as immune modulation or enhanced HPV activity [5,6,13,16,17,18]. However, neither HHV-8 nor HSV-2 DNA was detected in any of the samples in our study, including those with histologically confirmed premalignant lesions. These results are in agreement with earlier studies that reported low or undetectable levels of HHV-8 and HSV-2 DNA in cervical tissue [16,17,32].

This discrepancy may reflect regional differences in herpesvirus prevalence, differences in sample types (FFPE tissue versus cervical swabs), or limitations of diagnostic sensitivity [6,13,16]. Additionally, some studies that reported herpesvirus positivity were based on serological assays, which may not reflect the actual presence of viral DNA within the cervical tissue [17,18]. The neurotropic nature of HSV-2 and its ability to persist latently in ganglia may also contribute to the difficulty in detecting the virus in biopsy samples, particularly in asymptomatic individuals [33].

In line with recent molecular studies, our findings suggest that HHV-8 and HSV-2 are unlikely to play a significant role in the pathogenesis of premalignant cervical lesions [13,32,34]. Moreover, this finding is based solely on a single real-time PCR assay, which constitutes a methodological limitation and does not allow for broad generalizations regarding the absence or clinical insignificance of these viruses. Furthermore, it remains unclear whether the absence of herpesvirus DNA reflects true viral absence, technical limitations, or low regional prevalence. The sensitivity of PCR in FFPE samples, especially for latent viruses like HSV and HHV-8, may be insufficient to detect low-copy targets. Therefore, the inclusion of complementary approaches, such as mRNA-based detection assays or cervical swab sampling, could enhance diagnostic sensitivity. Additionally, incorporating epidemiological data on herpesvirus seroprevalence in the Turkish population would help contextualize these findings and improve the interpretation of viral absence in cervical tissues.

Nevertheless, the association between HSV-2 and cervical disease remains controversial. Some molecular and epidemiological studies suggest that HSV-2 might facilitate HPV persistence and activity through immune modulation or epithelial damage [35,36,37,38]. In contrast, other studies report no substantial contribution of HSV-2 to high-grade lesions or cervical cancer [19,28,39]. In our study, HSV-2 DNA was undetectable in all samples, which aligns with recent evidence indicating its negligible role in cervical carcinogenesis. This likely reflects its neurotropic nature, low cervical tissue load, and latent behavior [19,39]. Although HSV-2 may act as a cofactor in certain contexts, current data including ours do not support the direct or consistent role of HSV-2 in the development of premalignant cervical lesions.

In the present study, HSV-1 DNA was identified in only 1.1% of cases, all of which exhibited non-dysplastic histology. This limited detection, confined exclusively to histologically normal tissues, likely reflects a latent or incidental presence of HSV-1 in the cervical epithelium rather than a causal role in neoplastic transformation. Previous studies have similarly found no significant association between HSV-1 and cervical dysplasia or carcinoma, supporting its negligible role in cervical pathogenesis [6,39,40,41].

The absence of HSV-1 in premalignant or malignant lesions, combined with stringent laboratory protocols, reduces the likelihood of technical contamination. However, the lack of concurrent serological data prevents complete exclusion of transcriptionally inactive latent infections. These findings highlight the importance of integrating both molecular and serological approaches in future studies investigating herpesvirus co-infections in cervical disease.

To our knowledge, this study is the first in Türkiye to concurrently evaluate HR-HPV, HHV-8, and HSV-2 DNA in FFPE cervical biopsy samples classified by histopathology. The integration of molecular detection methods with histopathological diagnosis enabled a thorough analysis of the presence and potential contribution of these viruses within tissue samples.

Nonetheless, certain limitations should be acknowledged. DNA quality in FFPE samples may be partially degraded, which can reduce PCR sensitivity, especially for low-copy viruses such as HHV-8 and HSV-2 [13,42]. Furthermore, the PCR assay employed allowed full genotyping only for HPV-16, -18, and -45, while other high-risk genotypes were reported in grouped categories. This approach may have limited detection of full genotype diversity [21,22]. Additionally, the absence of cervical swab samples may have limited the detection of transient or superficial infections not established in tissue. Finally, the single-center nature of the study may affect the generalizability of the findings.

Despite these limitations, the use of FFPE specimens allowed for direct molecular-pathological correlation and provided useful data for retrospective evaluation. Our findings strengthen the evidence for the primary role of HR-HPV in cervical carcinogenesis and suggest that coinfections with herpesviruses do not appear to play a notable clinical role in this setting.

The observed 40.6% prevalence of HR-HPV in this study is consistent with previous studies conducted in similar populations. These studies support that HPV detection rates in biopsy specimens, particularly in high-grade lesions, are higher compared to cytological methods, such as smears, and increase with lesion severity. The main reason for this is that women undergoing biopsy generally represent a high-risk group, selected due to abnormal cytology, positive HPV testing, or suspicious lesions on colposcopy. Furthermore, biopsies are taken directly from the lesion and sample deeper tissue layers, which increases the likelihood of detecting HPV. Finally, the use of highly sensitive molecular tests can even detect latent infections, thereby contributing to the higher detection rates [43,44,45].

Taken together, HR-HPV was confirmed as the primary viral agent associated with pre-malignant cervical lesions, with a significant correlation between HPV DNA positivity and histological severity, particularly in HSIL and LSIL samples. HPV-16 remained the most prevalent genotype overall, while multiple HPV infections were more frequent among younger women and those with high-grade lesions.

However, an important limitation of this study is the restricted genotyping capacity of the real-time PCR kit used, which enabled full genotype identification only for HPV-16, -18, and -45. All other HR genotypes were grouped into pooled detection panels (for example, HPV-35/52, HPV-33/51/59), preventing discrete genotype-level analysis. This limitation hinders the ability to assess the individual contribution of specific genotypes beyond the three fully typed ones. Future studies should employ extended genotyping platforms, such as next-generation sequencing or type-specific multiplex assays, to achieve a more comprehensive and clinically informative HPV genotype profile.

The findings of this study are highly consistent with HPV genotype distributions reported in other regional studies conducted across Türkiye. HPV-16 and -18 have been identified as the most prevalent genotypes in various geographic areas, while other HR-HPV types have also been detected at notable frequencies [46,47]. Moreover, the association between multiple HPV infections and high-grade cervical intraepithelial neoplasia has similarly been demonstrated in several other studies [48].

In addition, large-scale multicenter analyses from Türkiye have revealed that non-16/18 HR-HPV genotypes constitute the majority of infections and are also significantly associated with HSIL and CIN2+ lesions, underscoring the need for broader genotyping strategies in screening and surveillance programs [49]. These cumulative data support the importance of including a wider range of high-risk genotypes when interpreting HPV-related cervical pathology, especially in regions with diverse genotype prevalence patterns.

These findings indicate that, especially in regions with diverse genotype distributions, it is essential to consider a broader range of high-risk genotypes when evaluating HPV-related cervical pathology. In Türkiye, HPV vaccination coverage among women has been reported to be as low as 3.6% [50], which underscores the public health significance of these results and highlights the urgent need for more accessible, publicly funded vaccination programs and awareness campaigns to reduce the burden of HPV-related cervical disease.

In contrast, HHV-8 and HSV-2 DNA were not detected in any case, and HSV-1 DNA was found only in a few non-dysplastic samples, with no evidence of co-infection with HPV. The use of FFPE tissue introduces another limitation, as DNA degradation, due to formalin fixation and long-term storage, may reduce detection sensitivity, particularly for viruses present at low copy numbers.

Formalin causes cross-linking between nucleic acids and proteins, as well as fragmentation and base modification of DNA, which can compromise the efficiency of PCR amplification. These chemical and physical changes particularly affect the detection of low-copy viral genomes or degraded targets, increasing the likelihood of false-negative results. Sensitivity may also be reduced for assays targeting longer amplicons or non-replicating viruses such as latent herpesviruses. To mitigate these limitations, future studies may benefit from targeting shorter PCR regions, using fresh/frozen samples, or applying confirmatory methods such as sequencing or nested PCR [22,42,51].

Additionally, relying solely on real-time PCR without cross-validation by conventional PCR or sequencing may restrict confirmatory accuracy. Lastly, the absence of clinical or serological data regarding prior herpesvirus exposure further limits interpretation, especially for latent viruses, such as HSV and HHV-8, which may not be detectable in tissue samples but still play a modulatory role in cervical pathogenesis. Although therapeutic vaccines for varicella–zoster virus (VZV) have shown clinical success, no licensed therapeutic vaccines currently exist for HSV, CMV, or EBV. This highlights the need for continued research into immunotherapeutic strategies for chronic herpesvirus infections [52].

## 5. Conclusions

HR-HPV DNA was detected in 40.6% of cervical biopsy samples, with the highest prevalence observed in women aged 30–39 years. These findings support the role of high-risk HPV infections, especially HPV-16, in the development of cervical lesions. HR-HPV positivity was significantly associated with increasing histological severity, particularly in LSIL and HSIL lesions. However, the absence of HHV-8 and HSV-2 detection in this study, based on a single real-time PCR assay, limits the ability to draw generalized conclusions regarding their clinical relevance. The lack of herpesvirus DNA in cervical tissues suggests only a minor role for these viruses in the progression of cervical lesions. Further large-scale, multicenter studies using both tissue-based and serological approaches is needed to validate these findings and to better elucidate the dynamics of viral co-infections in cervical disease.

## Figures and Tables

**Table 1:** Distribution of high-risk human papillomavirus (HR-HPV) infection and genotype patterns by age group.

	Age Groups (Years)					*p* Value
	30–39	40–49	50–59	≥60	Total	
HR-HPV genotype	n = 86	n = 91	n = 60	n = 39	n = 276	
Negative	42 (48.8)	51 (56.0)	39 (65.0)	32 (82.1)	164 (59.4)	0.004
Positive	44 (51.2)	40 (44.0)	21 (35.0)	7 (17.9)	112 (40.6)	
Single infection	18 (20.9)	18 (19.8)	12 (20.0)	4 (10.3)	52 (18.8)	
HPV 16	16 (18.6)	17 (18.7)	9 (15.0)	4 (10.3)	46 (16.7)	
HPV 18	2 (2.3)	1 (1.1)	3 (5.0)	0 (0.0)	6 (2.2)	
Multiple infection	20 (23.3)	12 (13.2)	4 (6.7)	0 (0.0)	36 (13.0)	
HPV 35/52, HPV 33/51/59	4 (4.7)	8 (8.8)	0 (0.0)	0 (0.0)	12 (4.3)	
HPV 16, HPV 18	6 (7.0)	1 (1.1)	1 (1.7)	0 (0.0)	8 (2.9)	
HPV 16, HPV 35/ 52	4 (4.7)	1 (1.1)	2 (3.3)	0 (0.0)	7 (2.5)	
HPV 16, HPV 18, HPV 35/52	0 (0.0)	1 (1.1)	0 (0.0)	0 (0.0)	1 (0.4)	
HPV 18, HPV 35/52	3 (3.5)	0 (0.0)	1 (1.7)	0 (0.0)	4 (1.4)	
HPV 16, HPV 45	2 (2.3)	1 (1.1)	0 (0.0)	0 (0.0)	3 (1.1)	
HPV 16, HPV 33/51/59	1 (1.2)	0 (0.0)	0 (0.0)	0 (0.0)	1 (0.4)	
Untypable	6 (7.0)	10 (11.0)	5 (8.3)	3 (7.7)	24 (8.7)	
HPV 35/52	4 (4.7)	5 (5.5)	5 (8.3)	1 (2.6)	15 (5.4)	
HPV 33/51/59	2 (2.3)	5 (5.5)	0 (0.0)	2 (5.1)	9 (3.3)	

HR-HPV, high-risk human papillomavirus. Untypable: HPV detected but genotype not determined. Data are presented as number (n) and percentage (%).

**Table 2:** Distribution of high-risk human papillomavirus (HR-HPV) genotypes according to histopathological diagnosis.

HR-HPV DNA	Normal/ Non-Dysplastic	LSIL	HSIL	Total	*p* Value
	n = 177	n = 50	n = 49	n = 276	
Negative	156 (88.1)	6 (12.0)	2 (4.1)	164 (59.4)	<0.001
Positive	21 (11.9)	44 (88.0)	47 (95.9)	112 (40.6)	
Single-type infection	11 (6.2)	24 (48.0)	17 (34.7)	52 (18.8)	
HPV 16	9 (5.1)	21 (42.0)	16 (32.7)	46 (16.7)	
HPV 18	2 (1.1)	3 (6.0)	1 (2.0)	6 (2.2)	
Multiple-type infection	2 (1.1)	9 (18.0)	25 (51.0)	36 (13.0)	
HPV 35/52, HPV 33/51/59	0 (0.0)	0 (0.0)	12 (24.5)	12 (4.3)	
HPV 16, HPV 18	1 (0.6)	6 (12.0)	1 (2.0)	8 (2.9)	
HPV 16, HPV 35/ 52	0 (0.0)	1 (2.0)	6 (12.2)	7 (2.5)	
HPV 16, HPV 18, HPV 35/52	0 (0.0)	0 (0.0)	1 (2.0)	1 (0.4)	
HPV 18, HPV 35/52	0 (0.0)	2 (4.0)	2 (4.1)	4 (1.4)	
HPV 16, HPV 45	0 (0.0)	0 (0.0)	3 (6.1)	3 (1.1)	
HPV 16, HPV 33/51/59	1 (0.6)	0 (0.0)	0 (0.0)	1 (0.4)	
Untypable	8 (4.5)	11 (22.0)	5 (10.2)	24 (8.7)	
HPV 35/52	7 (4.0)	6 (12.0)	2 (4.1)	15 (5.4)	
HPV 33/51/59	1 (0.6)	5 (10.0)	3 (6.1)	9 (3.3)	

LSIL: Low grade squamous intraepithelial lesions, HSIL: High grade squamous intraepithelial lesions. Untypable: HR-HPV detected but genotype not determined. Data are presented as number (n) and percentage (%).

**Table 3:** Association of high-risk human papillomavirus (HR-HPV) status, age groups, and infection patterns with cervical lesion severity (LSIL/HSIL) based on binary logistic regression analysis.

	Normal/Non Displastic	LSIL, HSIL	OR	95% CI	*p* Value
	n	n			
**HR-HPV status**					
Negative	156	8	Reference		
Positive	21	91	84.5	35.96–198.57	<0.001
**Genotype**					
HPV-negative	156	8	Reference		
HPV 16	9	37	80.17	28.98–221.78	<0.001
HPV 18	2	4	39.0	6.19–245.59	<0.001
HPV 16, HPV 18	1	7	136.5	14.94–1247.43	<0.001
HPV 35/52	7	8	19.5	5.5–69.15	<0.001
HPV 33/51/59	1	8	156.0	17.34–1403.63	<0.001
**Age groups**					
30–39	6	38	Reference		
40–49	8	32	1.06	0.26–4.31	0.928
50–59	6	15	1.36	0.23–8.17	0.734
≥60	1	6	1.12	0.10–12.15	0.928
**Infection pattern**					
Single	11	41	Reference		
Multiple	2	34	4.56	0.95–22.0	0.059
Untypable	8	16	0.54	0.18–1.58	0.258

Note: n: number; LSIL: low-grade squamous intraepithelial lesion; HSIL: high-grade squamous intraepithelial lesion; OR: odds ratio; CI: confidence interval. Reference groups were HPV-negative individuals, age group 30–39 years, and those with single HPV infection. The values presented under the “Age groups” and “Infection pattern” sections reflect only the distribution of HPV-positive cases within the “Normal/non-dysplastic” and “LSIL/HSIL” categories. Binary logistic regression analysis was used to assess the independent association between cervical lesion severity (presence of LSIL/HSIL) and age, HR-HPV infection status, and infection pattern.

## Data Availability

Data supporting the findings of this study are available from the corresponding author, Dr. Ayfer Bakır, upon reasonable request.

## Abbreviations

The following abbreviations are used in this manuscript:

HPV	Human papillomavirus
HR-HPV	High-risk human papillomavirus
HSV-1	Herpes simplex virus 1
HSV-2	Herpes simplex virus 2
HHV-8	Human herpes virus-8
FFPE	Formalin-fixed paraffin-embedded
LSIL	Low-grade squamous intraepithelial lesion
HSIL	High-grade squamous intraepithelial lesion
